# Phytochemical Properties, Extraction, and Pharmacological Benefits of Naringin: A Review

**DOI:** 10.3390/molecules28155623

**Published:** 2023-07-25

**Authors:** VS Shilpa, Rafeeya Shams, Kshirod Kumar Dash, Vinay Kumar Pandey, Aamir Hussain Dar, Shaikh Ayaz Mukarram, Endre Harsányi, Béla Kovács

**Affiliations:** 1 Department of Food Technology & Nutrition, Lovely Professional University, Phagwara 144001, Punjab, India; 2 Department of Food Processing Technology, Ghani Khan Choudhury Institute of Engineering and Technology Malda, Malda 732141, West Bengal, India; 3 Department of Bioengineering, Integral University, Lucknow 226026, Uttar Pradesh, India; 4 Department of Biotechnology, Axis Institute of Higher Education, Kanpur 209402, Uttar Pradesh, India; 5 Department of Food Technology, Islamic University of Science and Technology, Awantipora 192122, Kashmir, India; 6 Faculty of Agriculture, Food Science and Environmental Management Institute of Food Science, University of Debrecen, 4032 Debrecen, Hungary; 7 Faculty of Agriculture, Food Science and Environmental Management, Institute of Land Utilization, Engineering and Precision Technology, University of Debrecen, 4032 Debrecen, Hungary

**Keywords:** naringin, flavonoid, extraction, bioactive potential, pharmaceutical

## Abstract

This review describes the various innovative approaches implemented for naringin extraction as well as the recent developments in the field. Naringin was assessed in terms of its structure, chemical composition, and potential food sources. How naringin works pharmacologically was discussed, including its potential as an anti-diabetic, anti-inflammatory, and hepatoprotective substance. Citrus flavonoids are crucial herbal additives that have a huge spectrum of organic activities. Naringin is a nutritional flavanone glycoside that has been shown to be effective in the treatment of a few chronic disorders associated with ageing. Citrus fruits contain a common flavone glycoside that has specific pharmacological and biological properties. Naringin, a flavone glycoside with a range of intriguing characteristics, is abundant in citrus fruits. Naringin has been shown to have a variety of biological, medicinal, and pharmacological effects. Naringin is hydrolyzed into rhamnose and prunin by the naringinase, which also possesses l-rhamnosidase activity. D-glucosidase subsequently catalyzes the hydrolysis of prunin into glucose and naringenin. Naringin is known for having anti-inflammatory, antioxidant, and tumor-fighting effects. Numerous test animals and cell lines have been used to correlate naringin exposure to asthma, hyperlipidemia, diabetes, cancer, hyperthyroidism, and osteoporosis. This study focused on the many documented actions of naringin in in-vitro and in-vivo experimental and preclinical investigations, as well as its prospective therapeutic advantages, utilizing the information that is presently accessible in the literature. In addition to its pharmacokinetic characteristics, naringin’s structure, distribution, different extraction methods, and potential use in the cosmetic, food, pharmaceutical, and animal feed sectors were discussed.

## 1. Introduction

Numerous phytochemicals, such as flavonoids (such as hesperidin and naringin), limonoids (such as limonin and nomilin), carotenoids (such as beta-carotene and lutein), and vitamin C are abundant in citrus fruits. Citrus fruits’ vivid colors, distinctive flavors, and distinctive scents are all influenced by these phytochemicals. Citrus fruits include a variety of phytochemicals that have many health advantages [1]. They have antioxidant capabilities that assist the body in fighting off dangerous free radicals and guarding against oxidative stress and cellular damage. Citrus phytochemicals have also been associated with anti-inflammatory effects, which can help reduce the risk of chronic diseases like cardiovascular disease and certain types of cancer. Citrus fruit polyphenols have also been linked to stronger immune systems, better cardiovascular health, and potential anti-diabetic effects. According to some research, these substances may assist with healthy weight management, lowering cholesterol levels, and lowering blood pressure. Citrus fruits include a wide variety of phytochemicals that are essential for overall health and wellbeing, thus including them in the diet is crucial. Beyond what can be achieved by a single vitamin, these chemicals act synergistically to promote health. Regular citrus fruit consumption can support optimal health and lower the risk of chronic diseases by promoting a balanced and nutrient-rich diet [2].

Citrus species are among the most frequently cultivated fruit crops around the world, used to make both fresh juice and food products. The plant genus Citrus, which comprises several varieties of oranges, sour and sweet oranges, tangors, lemons, and tangerines, is a member of the Rutaceae family [3]. Citrus fruits are an excellent supplier of secondary metabolites like terpenoids and polyphenols. These are rich in vitamins A, vitamin C, vitamin E, dietary fibers, and essential minerals [4]. Natural phenolic molecules called flavonoids have a wide variety of bioactivities. Three rings, including two benzene rings and 15 carbon atoms, make up the basic flavonoid structure [5]. Any flavonoid’s antioxidant potential is decided by the presence of hydroxyl groups in positions 3,5, an O-dihydroxy structure in the B-ring, a 2,3-double bond conjugated with 4-oxo function, and a 2,3-double bond. A total of 4000 flavonoids have already been isolated, mostly in fruits, herbs, and vegetables. The concentrations and profiles of citrus flavonoids differ greatly between species [6].

The peels, pulp, seeds, and juice of citrus fruits contain a variety of bioactive compounds [1]. Citrus peel has a wealth of bioactive chemicals, including natural antioxidants like flavonoids, and accounts for 50 percent to 65 percent of the total mass of the fruits [7] Citrus flavonoids that have been isolated were discovered to have anti-inflammatory, anti-bacterial, anti-aging, anti-cancer, cardiovascular protective, and hepatoprotective properties in several investigations [8]. Naringin, scientifically known as 5,7-trihydroxyflavonone-7-rhamnoglucoside, comes under the category of flavanone glycoside and it is found in grapes and citrus fruits. The quantity of naringin in fruit is usually determined by its maturity. The immature fruit has a higher concentration of naringin. The fruit maturity is an essential consideration in juice processing, particularly in grapefruit juices, which have a high level of bitterness [9].

Numerous scientific studies have stated that naringin alters the blood levels of some medications, when taken concurrently, by interfering with the operations of enzymatic proteins and transporters in the intestines [10]. Naringin is a powerful inhibitor of transporter proteins such as the multidrug resistance protein (MDR) and organic anion transporting polypeptide (OATP) isoforms, as well as sulfotransferase (SULT). Naringin inhibits a number of cytochrome isoenzymes (CYP) as well [11]. It has been demonstrated that the anticancer medication naringin reduces the expression of p-glycoprotein, which is a membrane-associated drug efflux pump whose increased expression causes anticancer medication resistance for doxorubicin. The flavanone naringin also prevents CYP isoenzymes by inhibiting the production of carcinogens, indicating a potential role in the mitigation of cancer [12]. The bitterness brought by naringin in the manufacturing of commercial grapefruit juice can be removed using a specific enzyme known as naringinase. Two rhamnose units are attached to its aglycon portion, naringenin, at the 7-carbon position. Both naringin and naringenin are strong antioxidants [13]. Naringin is less potent compared with naringenin because the sugar moiety in the former causes steric hindrance of the scavenging group. Naringin is moderately soluble in water. The gut microflora breaks down naringin to its aglycon naringenin in the intestine; it is then absorbed from the gut [1]. However, the enzyme naringinase, which is present in the stomach of humans, transforms naringin into aglycone naringenin. The objective of this review was to assess the various innovative approaches implemented for the extraction of naringin and to study its chemical structure, chemical components, and potential food sources. The pharmacological properties of naringin, including its potential as an anti-diabetic, anti-inflammatory, and hepatoprotective substance, were also discussed. This review also explored the wide range of biological, medicinal, and pharmacological effects associated with naringin along with its applications in various industries such as cosmetic, food, pharmaceuticals and animal feed [14].

## 2. Chemical Composition of Naringin

The flavonoid substance naringin is mostly present in grapefruits and other citrus fruits. In chemical terms, it is a glycoside made up of the disaccharide neohesperidose and the flavone naringenin. The chemical structure of naringin consists of a flavonoid backbone, two phenolic rings, and a heterocyclic pyran ring. Its molecular weight per mole is 580.54 g and its chemical formula is C_27_H_32_O_14_. Pharmaceutical and nutraceutical research is interested in naringin because of its bitter taste and its variety of biological qualities, such as antioxidant, anti-inflammatory, anticancer, and cardioprotective properties [1].

### 2.1. Significance of Flavonoids

In plants, animals, and microbes, flavonoids have a variety of biological effects. Long known to be synthesized at specific locations in plants, flavonoids are also important for the color and scent of flowers, the ability of fruits to draw pollinators and, as a result, fruit dispersion, the germination of seeds and spores, and the development and growth of seedlings. Plants are protected from various biotic and abiotic challenges by flavonoids, which also serve as special UV filters, allopathic substances, signal molecules, phytoalexins, antimicrobial defensive components, and detoxifying agents. Flavonoids have protective effects against frost drought resistance and hardiness, and they may serve to help plants adapt to heat and tolerate freezing temperatures. There are six types of flavonoids [14]. The major classes of flavonoids, their examples, chemical structures, and main dietary sources are listed in Table 1.

Flavones, which are distinguished by a flavone backbone, are often present as glucosides in a variety of fruits and vegetables, including parsley, celery, chamomile, red peppers, and mint [20,21]. Proanthocyanins are created from flavonols, which are found in large quantities in a variety of foods such as tomatoes, grapes, apples, and berries. Flavonols are identified by a ketone group and a hydroxyl group in the third position of the C ring [22,23,24]. Isoflavones have a flavone-like structure and are mostly found in legumes like soybeans and leguminous plants. They are frequently found as glycosides, which the gut bacteria can change into aglycones [3,25]. Anthocyanins are phenolic chemicals that are members of the flavonoid family and make up the biggest category of water-soluble pigments. Flowers, plants, and fruits are given bright colors by them, which are distinguished by a flavylium cation structure [18,26]. Citrus fruits including lemons, oranges, and grapes contain flavanones, which are dihydroflavones with an unsaturated C ring [27]. Flavanols, often referred to as flavan-3-ols or flavanonols, are substances that have a hydroxyl group in the third position of the C ring on the flavan skeleton. These substances, which provide a wide range of health advantages, are found in many different plant-based sources [24,28].

The two primary groups of phenolic chemicals present in citrus fruits are flavonoids and phenolic acids. Citrus flavonoids have been proven to have anti-cancer, anti-inflammatory, anti-aging, anti-bacterial, hepatoprotective, and cardiovascular protective effects, according to several studies. The primary class of phytochemicals found in citrus fruits, particularly in the pulp, peels, and seeds, are called flavonoids. Flavones, flavanones, and flavonols are the three main categories of citrus flavonoids. Table 2 depicts the classification of citrus flavonoids isolated in citrus species, major fruit sources, C-ring structures, and substitution patterns [29].

### 2.2. Structure of Naringin

**Figure 1:**
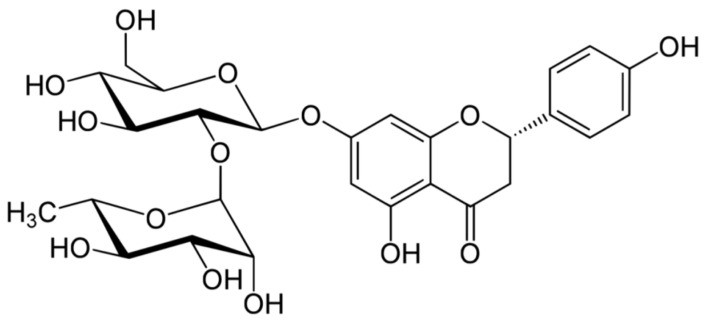
Molecular structure of naringin.

Asahina and Inubuse identified and characterized the chemical structure and molecular formula of naringin in 1928. A 2-O-(alpha-L-rhamnopyranosyl)-beta-D-glucopyranosyl moiety is substituted at position 7 of the disaccharide derivative naringin by an alpha-L-rhamnopyranosyl group via a glycosidic bond. The melting point of naringin is 83 °C at a solubility of 1 mg/mL at 40 °C and the molecular weight of naringin is 580.5 g/mol [24]. The molecular structure of naringin is shown in Figure 1. With a rise in temperature, naringin and naringenin, its aglycon equivalent, become more soluble in various solvents. In the order of methanol, ethyl acetate, n-butanol, isopropanol, petroleum ether, and hexane, naringin was soluble in the six solvents [30]. Naringin complexes are 15 times more soluble in water at 37 ± 0.1 °C than free naringin. It starts to degrade at temperatures above 100 °C or when light is present [31]. The presence of a carboxylic group is suggested by the wide, strong -OH stretching absorption from 3300 per cm to 2500 per cm. Alcohols and phenols are represented by the strong and wide hydrogen-bonded O-H stretching bands centered at 3300 cm^−1^ and 3400 cm^−1^ [17]. The C=C stretching bands for aromatic rings typically appear outside the typical region where C=C emerges for alkenes (1650 cm^−1^) between 1600 and 1450 cm^−1^. These peaks only occur with naringin. When researching flavonoid-cyclodextrin inclusion complexes, it was discovered that the characteristic peaks for aromatic rings and phenols in naringin at 1519 cm^−1^ and 1361 cm^−1^ had disappeared. The amount of naringin measured and the correlation coefficient (r) for sensory bitterness was 0.97IBU [32].

## 3. Sources of Naringin

**Figure 2:**
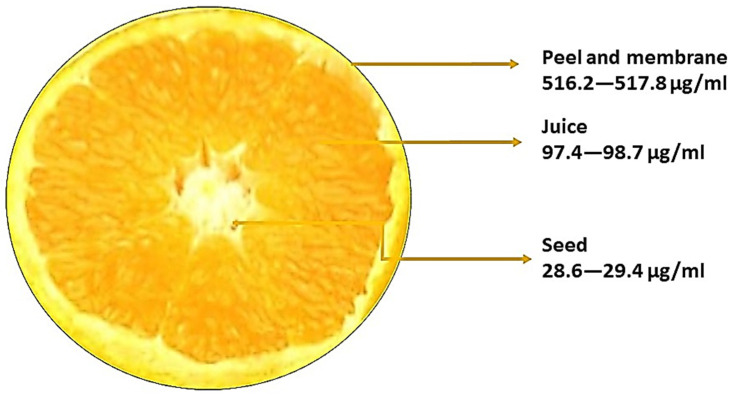
The distribution of naringin in *Citrus aurantiifolia*.

Plants contain a variety of flavonoids, which are widely dispersed and have significant biological functions. Since the quantity of naringin is comparatively higher at the immature stage, citrus fruits are typically used in studies to determine the amount of naringin in fruits [33]. Citrus fruits provide a large number of flavonoids in the diet. Naringin is mostly found in the peel of grapefruit, lime, and their variations; it has several biological functions and is frequently used in food, cosmetics, and medicine. Naringin is a glycoside flavanone seen in grapes and citrus fruits. Naringin was first discovered by DeVry in 1857 [34]. It has been reported that the pith contains a higher quantity of naringin in grapefruit, followed by the peel with the membrane, the seeds, and the juice [35]. The amount of naringin in the seeds of grape fruit is 200 μg/mL and 2300 μg/mL is found in the peel of grape fruit [36]. Pummelo has plenty of naringin in it. Compared to the juice, the quantity of naringin was higher in the peel; the naringin content of the juice of pummelo is 220 μg/mL and in the peel it is 3910 μg/mL. The amount of naringin in lime is very low when compared with pummelo. In both species, a high amount of naringin content is present in the skin of the fruits. The amount of naringin found in skin, juice, and seed is 517.2 μg/mL, 98 μg/mL, and 29.2 μg/mL, respectively [37]. The distribution of naringin based on the calculations of various studies in *Citrus aurantiifolia* is shown in Figure 2. Naringin content in sour orange is 47.1 μg/mL. In sour orange flower, the amount of naringin in the receptacle, ovary, and stigma is 1.3444 μg/mL, 9.036 μg/mL, and 2.554 μg/mL, respectively [38]. Phenol is a chemical compound with a hydroxyl group attached to an aromatic ring. Tannin is a type of phenol compound found in plants, known for its astringent properties. Naringin is a flavonoid compound found in citrus fruits that exhibits antioxidant and anti-inflammatory effects.

## 4. Extraction of Naringin

**Figure 3:**
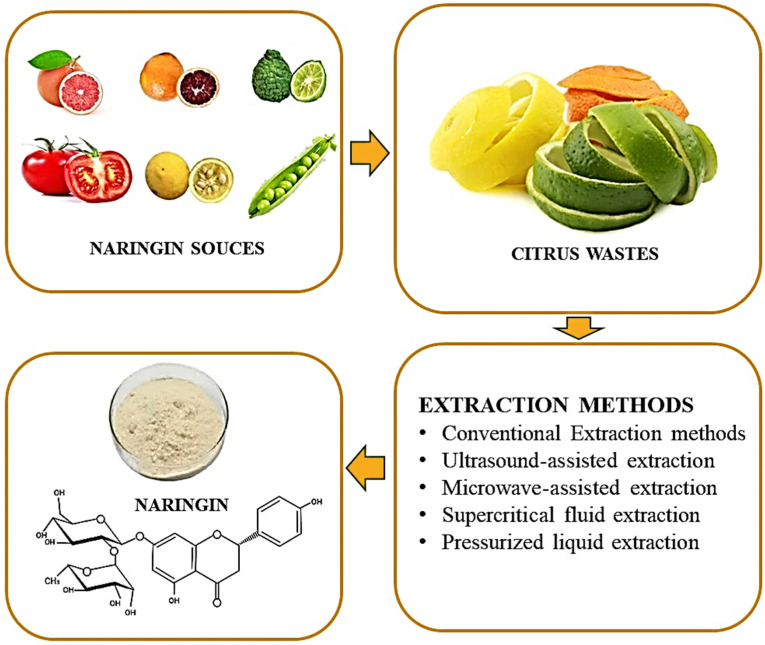
Extraction of naringin using different techniques.

The citrus peel contains significant levels of the flavanones neohesperidin, hesperidin, naringin, and narirutin as well as polymethoxylated flavones tangeretin, sinensetin, and nobiletin. Flavonols, glycosylated flavones, and hydrocinnamic acid are present in very minor amounts [39]. There are three main steps for naringin isolation from fruits including extraction, separation, and purification [29]. Only after utilizing the proper extraction process can flavonoids be isolated, recognized, and classified. The amount of naringin in fruit is determined by numerous factors which include the harvesting time of the fruit, the section of fruit utilized, and whether the peel is a source of naringin. The most prevalent method of extraction is conventional solvent extraction [40]. The various non-traditional techniques include high hydrostatic pressure extraction [41], ultrasound assisted extraction [42], microwave assisted extraction [43], and subcritical [44] and supercritical [45] extraction. The first step in the process involves pre-treating or preparing the sample, during which centrifugation, filtration, drying, and other techniques may be performed. Naringin is extracted, isolated, and purified from various plant materials in the second stage. Naringin is extracted utilizing techniques like soxhlet, maceration, water infusion, microwave extraction, ultrasound extraction, supercritical fluid extraction, auto-hydrolysis, and solid micro-phase extraction, etc. in this step, as given in Figure 3. The final phase often involves the identification, quantification, and recovery of flavonoid components using chromatography techniques on the purified and extracted extracts.

### 4.1. Conventional Techniques for Naringin Extraction

The most extensively used conventional method for extracting flavonoids is liquid–liquid or solid–liquid extraction. For the extraction of bioactive chemicals, these approaches have incorporated the use of solvents such as ethanol (C_2_H_6_O), methanol (CH_3_OH), and acetone (CH_3_)_2_CO rather than only water. Because of the significantly greater yields obtained in the recovery of flavonoids, ethanol (C_2_H_6_O) and methanol (CH_3_OH) are the most extensively utilized solvents for flavonoid extraction [40]. In a study, soxhlet method was used for extraction on fresh grapefruit peel samples; 40.0 g was grounded for 1 min in a blender with 100 mL Ethyl alcohol before the mixture was filtered and the filtrates and residue were separated. For removing the alcohol, the residue was air dried. A portion of the filtrate was put in a solvent extraction flask with 50 mL of ethanol and extraction was completed in three hours. After that, the filtrate was evaporated and dried at 50 °C and kept at room temperature.

### 4.2. Novel Techniques for Naringin Extraction

Numerous innovative extraction techniques have been developed to provide more environmentally friendly extraction processes that utilize the minimum amount of energy and solvent by generating high amounts of yields. Many of these techniques made use of accelerators like microwave or ultrasound, as well as supercritical fluid or subcritical fluid and the use of high pressure. To accelerate the extraction operation and improve the extraction kinetics and yield, several authors recommended using two processes in sequence, like ultrasonic aided extraction and instant controlled pressure drop technology, or a combination of techniques, like enzyme-assisted extraction [20].

Naringin from the peel of *Citrus paradisi* L. can be extracted using supercritical fluid extraction (SFE) techniques with the highest yield of 14.4 g/kg. In this technique, SC modified with 15% ethanol and fresh peels at 1377.86 psi and 58.6 °C can also be used which will result in a lower consumption of solvent and time (45 min) [42]. Naringin from grapefruit seeds has been extracted using the supercritical fluid extraction process. For this, the extraction process was split into two halves. SC-CO_2_ was used to extract less polar limonin in stage one, whereas SC-CO_2_ was modified with C_2_H_6_O as a co-solvent in stage two for the extraction of high polar limonin-17-b-D-glucopyranoside (LG) and naringin. The highest quantity of naringin, which is 0.2 mg/g from seeds, was obtained with the optimized conditions of 40 min at 41.4 MPa pressure, 50 °C temperature, and 20% ethanol concentration. The flow rate of the given mobile phase was kept constant at 5.0 L/min throughout the trials. The results showed that SC-CO_2_ extraction of naringin from grapefruit seeds is an ecologically friendly and feasible method [46].

Ultrasonic extraction was found to be effective for extracting naringin from ripe pomelo peels. The effectiveness of this method was determined by the agent concentration, the sample-to-solvent ratio, and the ultrasonic duration. In this experiment, 0.6 L of 70% of aqueous ethanol was mixed with 80 g of powdered material in a flask. The flask was kept in the ultrasonic bath with the frequency of 0.04 MHz for 30 min, which was followed by a filtering process that was performed once more. The naringin concentration from peels of ripe *Citrus maxima* is 2.20 % and the yield of purified naringin is 77.26 % under optimal purification conditions, according to the data [4]. The ultrasound-assisted aqueous two-phase extraction (UA-ATPE) method is used for extracting synephrine, neohesperidin, and naringin from *Citrus aurantium* L. fruitlets and is also used for their preliminary purification. Five distinct forms of ethanol or salts from an aqueous two-phase system (ATPS) were used for response surface methodology (RSM) and single-factor studies to further tune the extraction conditions. The following are the optimal process parameters: 20.60% (*w*/*w*) K_2_CO_3_, 27% (*w*/*w*) ethanol, 45.17:1 (g:g) solvent to material ratio, the 120-mesh particle size of fruit powder, 50 °C temperature, 30 min extraction period, and 80 W ultrasonic power. Under the given conditions, the yields of naringin, synephrine, and neohesperidin were 7.39 mg/g, 11.17 mg/g, and 89.27 mg/g, respectively [46].

Deep eutectic solvents can also be considered promising green and efficient solvents for the extraction of naringin, hesperidin, and neohesperidin from citrus fruits such as *Aurantii Fructus*. In a study, a series of tunable deep eutectic solvents was prepared and investigated by mixing choline chloride or betaine with different hydrogen-bond donors, and betaine/ethanediol was found to be the most suitable extraction solvent. The optimum extraction conditions were 40% of water in betaine/ethanediol (1:4) at 60 °C for 30 min extraction time with solid/liquid ratio 1:100 g/mL. Under these conditions, the extraction yield of narirutin, naringin, hesperidin, and neohesperidin was found to be 8.39 ± 0.61, 83.98 ± 1.92, 3.03 ± 0.35 and 35.94 ± 0.63 mg/g, respectively, which was comparatively higher than when using methanol as extraction solvent [47]. It has also been reported that deep eutectic solvents or aqueous glycerol can replace the traditional solvents for the extraction of polyphenols (naringin) from citrus peels such as grapefruit peels. It can increase the extraction of polyphenols and especially naringin flavonoid from grapefruit peels as compared to water [48].

## 5. Schematic Overview of the Possible Health Benefit Based on the Literature Review

**Figure 4:**
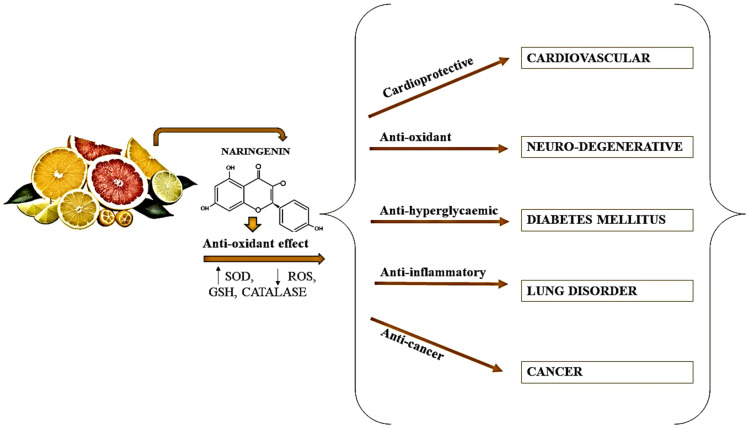
Potential health benefits of naringin; SOD: superoxide dismutase, ROS: Reactive oxygen species, GSH: γ-l-glutamyl-l-cysteinyl-glycine (glutathione).

Since ancient times, citrus fruits have been utilized as natural herbal treatments in traditional medicine. Citrus peel has been utilized in traditional Chinese medicine to enhance digestion, minimize gastric gas, bloating, and clear congestion [12]. Clinical and epidemiologic research states that eating citrus fruits lowers the risk of lifestyle-related disorders like cancer, cardiovascular disease, diabetes (type-2), and osteoporosis [49]. Naringin has been shown to have anticancer, antiapoptotic, cholesterol-lowering, antiatherogenic, and metal binding capabilities, as well as antioxidant qualities, as shown in Figure 4. Naringin is also said to enhance medication absorption and metabolism [50].

### 5.1. Anticancer Properties of Naringin

Naringin has been reported to inhibit many malignancies through the regulation of various cellular signaling cascades, including the inhibition of malignant cell growth, the induction of apoptosis and also the arresting of the cell cycle and the regulation of oxidative stress, inflammatory processes, and angiogenesis [51]. It was discovered that naringin at concentrations of 250–2000 M promoted cell apoptosis in cervical cancer cells (SiHa) in a dose-dependent way. This impact of naringin is thought to have contributed to the suppression of cell growth as well and also increase in apoptosis [29].

Naringin in the concentrations of 1 M, 5 M, and 10 M has reduced cell mortality caused by rotenone in human neuroblastoma cells (SH-SY5Y). In 4, 6-diamidino-2-phenylindol (DAPI) staining and terminal deoxynucleotidyl transferase dUTP nick end labeling (TUNEL) tests, naringin prevents condensation of chromatin and breakage of DNA strand production by rotenone [52]. Naringin also decreases rotenone-induced phosphorylation of the mitogene-activated kinase (MAPK) family members p38 and Jun NH2-terminal protein kinase (JNK) [53]. According to one study, naringin inhibits the growth of cells, and apoptosis was induced in K562, HL-60, and Kasumi-1 human myeloid leukemia cells in a concentration- and time-dependent manner by downregulating Mcl-1 expression and activating the caspase and PARP pathways. In U937 and THP-1 human leukemia cells, naringin therapy increased cell death and lowered cell cervical proliferation and expansion [54]. The most dangerous and prevalent brain tumors are gliomas, and they are still fatal despite advances in therapeutic care. As a result, a variety of therapy techniques are required to combat this deadly disease. In glioblastoma cells, naringin was able to block FAKp-Try397 and limit focal adhesion kinase (FAK) activity and its downstream pathway. Naringin treatment of U251 glioblastoma cells and U87 glioblastoma cells restricts their growth by inhibiting the cyclin D1 pathway or FAK pathway and induces the death of cells by inhibiting the BAD pathway or FAK pathway. By inhibiting the MMP or FAK pathways, it prevents metastasis of cells and invasion of cells. In the case of U251 glioma cells, naringin treatment restricts the proliferation of cells and its viability [55]. Naringin also inhibits the migration of cells and invasion of cells by modulating the matrix metallopeptidase-2 (MMP-2) expression and MMP-9. As a result, by decreasing the p38 signal transduction pathways, naringin has a potential and therapeutic effect on the regulation of invasive malignant gliomas [56].

Breast cancer is a term that refers to various types of cancers. A vast variety of individualized treatments for breast cancer have recently been offered, all of which have been shown to be effective [57]. Chemotherapy and cancer chemoprevention are both carried out with natural products containing bioactive chemicals. In MCF-7 cell lines, naringin treatment reduced proliferation and growth while also increasing apoptosis. In canine mammary cancer cells (CMT-U27), naringin oxime treatment decreased cell proliferation and viability [53]. Cervical cancer is the second most common cancer in women around the globe and continues to be difficult. At a dose of 750 M, naringin displayed a 50% suppression of SiHa human cervical cancer cells. Apoptosis, intra nucleosomal DNA fragmentation, morphological abnormalities, and mitochondrial transmembrane potential reduction were seen in SiHa cells. The findings imply that naringin works well in human cervical cancer treatment [58]. The common extra-cranial solid tumor in children is neuroblastoma. Plant-derived nutritional chemicals are gaining popularity as a treatment for a variety of solid tumors, including malignant neuroblastoma [59]. Naringin therapy reduced the viability of cells and induced apoptosis in rotenone-treated SH-SY5Y human neuroblastoma cells via inhibiting P38 and JNK phosphorylation and activating caspase-9 and caspase-3 [54]. The common malignant tumor in the endocrine system is thyroid cancer. In SW1736 cells, a TPC-1 naringin dose dependently raised the expression of Bax, cleaved Caspase3, and Caspase3, when the expression of c-Myc, cyclin D1, Bcl-2, and survivin decreased. It also decreased AKT pathway or P13K pathway activation in thyroid cancer (TC) cells. The naringin showed anti-tumor actions in TC cells by limiting cell division of TC and cell death promotion by controlling the gene expression associated with cell apoptosis and division and activating the AKT pathway or PI3K pathway [60].

### 5.2. Antidiabetic Properties of Naringin

It has been demonstrated that naringin enhances insulin sensitivity. Naringin has shown that it can improve insulin action and cell uptake of glucose. Insulin resistance is a major contributor to the onset of type 2 diabetes. This can enhance overall glycemic control and help control blood sugar levels. Insulin moves sugars from the bloodstream into cells, where they are used or stored as energy [61]. Diabetes is when the body does not produce enough insulin or cannot utilize the insulin it makes efficiently [5]. Diabetes is divided into two types. Type 1 Diabetes is a condition that is autoimmune. Cells in the pancreas, which make insulin, are attacked and destroyed by the immune system and what generates this attack remains an enigma. Approximately 10% of diabetics have this type of diabetes [32]. In type 2 diabetes, blood sugar levels increase if the body becomes resistant to insulin [62]. Some enzymes involved in the metabolism of carbohydrates may be inhibited by naringin. It has the ability to block glucosidase, an enzyme that converts complex carbs into simple sugars. Naringin can lower postprandial glucose levels by blocking this enzyme, which will slow down the digestion and absorption of carbohydrates. While naringin has the potential to be an effective anti-diabetic substance, it should not be used as a stand-alone treatment for diabetes. Along with traditional diabetes treatment techniques like a good diet, frequent exercise, and prescribed medications, it might be considered as a complimentary strategy.

Naringin is a powerful biomolecule that has the potential to help people with diabetes and its consequences [63]. Naringin restricts the secretion and sensitivity of insulin, PPAR, glucose transporters, blood lipids, hepatic glucose production, peripheral glucose uptake, intestinal glucose absorption, biosynthesis of cholesterol, oxidative stress, and inflammation [64]. Inflammatory cytokines are elevated and insulin resistance and hyperglycemia are generated by a high-fat diet. Naringin’s hypoglycemic impact has been thoroughly documented. Vitamin C (50 mg/kg) with naringin co-treatment improved insulin concentration and oxidative stress reduction in rats with streptozotocin-induced diabetes [5]. In diabetic nephropathic rats, naringin minimized streptozotocin-induced kidney dysfunction and damage, inhibited streptozotocin-induced oxidative stress in vivo, and prevented high glucose-induced apoptosis and ROS levels in vitro [65]. Naringin has anti-inflammatory and antioxidant benefits in diabetic nephropathic rats, as evidenced by the downregulation of IL-1, proinflammatory cytokines TNF, and IL-6 and the upregulation of antioxidants SOD, GSH, and CAT [6].

### 5.3. Anti-Inflammatory Properties of Naringin

The process by which the body’s white blood cells and the substances they make protect against bacterial and viral illness is known as inflammation. Flavanone-rich plants, such as naringin, hesperidin, and neohesperidin, have long been known to have anti-inflammatory properties [3]. Inflammation is divided into two types. Acute inflammation is the body’s reaction to a quick injury, such as cutting your finger. Your body sends inflammatory cells to the wound to help it heal. The healing process begins with these cells [66]. Chronic inflammation occurs when your body sends inflammatory cells even when there is no external threat. Inflammatory cells and chemicals assault joint tissues in rheumatoid arthritis, for example, causing an inflammation that comes and goes and can cause serious damage to joints, including pain and deformity [67].

The anti-inflammatory process controlled by nuclear factor-erythroid 2–related factor 2 (Nrf2) regulates cellular antioxidant synthesis and thus plays a very important role in preventing various degenerative illnesses [68]. In 3-nitropropionic acid-induced rats, naringin upregulates the expression of mRNA in HO-1, GST P1, NAD(P)H:quinone oxidoreductase 1, and g-glutamylcysteine ligase; this is followed by activating Nrf2 and the reduced expression of proinflammatory mediators like TNF-a, cyclooxygenase-2, and inducible NO synthase [69]. Naringin did not inhibit cell proliferation, but it did inhibit RANTES (regulated upon activation of normal T-cell expressed and secreted) production in a human epidermal keratinocytes cell line (HaCaT cells) by restricting nuclear translocation of NF-JB [70]. In animal models of inflammation, naringin has been demonstrated to reduce the production of inflammatory signaling factors like interleukin-8 (IL-8), interleukin-6 (IL-6), inducible nitric oxide synthase (iNOS), TNF-a, and nuclear factor erythroid 2-related factor 2 (Nrf2). Naringin treatment prevented an improvement in serum IL-6 during aging-related inflammation in 20-month-old male Wistar rats [54]. Naringin, neohesperidin, paeoniflorin, and platycodin-D are all found in “painopowder”, a traditional Chinese medicine. The four-ingredient combination had the greatest anti-inflammatory impact in a model of acute inflammation, while naringin was shown to have the most important role among the four substances [71].

### 5.4. Hepatoprotective Properties of Naringin

The capability of a chemical compound to inhibit liver toxicity is known as hepatoprotection [72]. Naringin is suggested to enhance the functioning of the hepatic antioxidant system as well as the metabolism of hepatotoxic substances [73]. Naringin exhibits protection against naturally occurring genotoxins in food, like PhIP (2-Amino-1-methyl-6-phenylimidazo[4,5-b] pyridine) and other cooked food mutagens, by lessening PHIP induced genotoxicity in human liver segments at a concentration of 1000 M [69]. Naringin (0.05–0.125 g/L) increased ethanol and lipid metabolism in rats, alleviating the adverse effects of ethanol consumption. It also reduced necrosis, steatosis, and fibrosis in rat models of alcoholic liver disease, as demonstrated by reduced expression of PGC1α (Peroxisome proliferator-activated receptor-gamma coactivator) or Sirt1; it is an enzyme involved in regulating energy metabolism in response to calorie restrictions at a dosage of 100 mg/day [71]. Table 3 describes various medical conditions and the therapeutic effect of the action of naringin.

### 5.5. Pharmacokinetics of Naringin

Studies were conducted with help of rats to understand the pharmacokinetic properties of naringin. The study of the absorption, distribution, metabolism, and excretion of drugs is known as pharmacokinetics [78]. Proton-coupled active transport and passive diffusion are used to absorb flavanone aglycones into the enterocytes. The low molecular weight, high lipophilicity, and slightly acidic character of aglycones cause passive diffusion. Once within the cells, naringin is expected to go through phase I metabolism, such as oxidation or demethylation by cytochrome P450 monooxygenases, then passing to phase II metabolism, such as sulfation, glucuronidation, or methylation, in intestinal cells or liver cells [79,80]. Naringin is rapidly absorbed in the blood, with the initial concentration peaking at 15 min and the second peaking at 3 h after naringin monomer oral administration; 480 min later, it is undetectable [81]. The affinity of these food chemicals for serum albumin, the primary transport protein, coincides with their tissue distribution and elimination. To begin with, the bound medication works as a reservoir and has a prolonged half-life, whilst the unbound portion is responsible for the biological impact. Second, drug binding to this protein reduces drug filtration by the kidney [82].

In terms of tissue distribution, the liver had the largest quantities of flavanone conjugates after repeated or single dose flavanone treatment in rats. By partially undergoing breakage of the bacterial ring and then the three bridges of carbon to dihydrochalcone moiety, naringin is eliminated by the kidneys into the urine and by the liver into bile According to Fuhr and Kummert’s findings (1995). Urine excretion ranges from 5 to 57 percent of total intake. Sulfates were the most common naringenin type detected in the tissues of rats. Only the liver and kidney had glucuronide concentrations that could be measured [83]. The average Cmax of naringin in portal plasma was 18.83.8 min (determined by the concentration reached at tmax in portal plasma), whereas the absorption ratios of naringin in portal plasma and lymph fluid were approximately 95.9 and 4.1, respectively, after naringin administration via a duodenal cannula (600 and 1000 mg/kg). This suggests that naringin is absorbed largely by portal blood rather than mesenteric lymph fluid and that it is excreted primarily by bile, with just a tiny quantity entering systemic circulation following hepatic metabolism [84]. Grapefruit juice, a key factor in pharmacokinetic drug interactions, possesses a higher amount of naringin, hence it was previously thought to be moderating these interactions. Despite extensive investigation, the constituents in grapefruit juice responsible for drug interaction, particularly P-glycoprotein and CYP3A4 levels, are unknown; however, it appears that certain coumarins, rather than flavanones, are likely involved [85]. However, naringin has also been shown to possess therapeutic properties and has potential as a therapeutic agent to treat numerous diseases such as various liver, heart, and metabolic disorders [86].

## 6. Application of Naringin

Naringin has many benefits for numerous industries including the food, pharmaceutical, cosmetics, and animal feed industries.

### 6.1. In Cosmetic Industry

The flavonoid naringin has anti-cancer, anti-oxidative, anti-aging, antibacterial, anti-inflammatory, cholesterol-lowering, and free radical scavenging properties [87]. Studies show that naringin reduces the risk of toxicity caused by other sunscreen ingredients like TiO_2_ when it is added to sunscreen formulations because of its antioxidant activity. It also scavenges free radicals produced by UV radiation and by the photocatalytic activity of ZnO and TiO_2_, which further lowers the risk of toxicity [88]. Essential oils like eucalyptus oil, lavender oil, and peppermint oil, are known to have strong antioxidant and antibacterial qualities that could be employed as environmentally friendly substitutes for synthetic antioxidants and preservatives in skin care formulations [70,89]. Naringin-loaded microemulsions were created using essential oils as the oil phase; these microemulsions showed antioxidant and antibacterial effects that were comparable to or outperformed those of synthetic ones. In comparison to their unformulated counterparts, naringin-loaded microemulsion-gel formulations demonstrated improved stability and release profiles. Skin care formulations have experienced advantages by utilizing microemulsions of essential oils. These microemulsions enhance the release and permeation of active ingredients into the skin, improve their stability, and serve as environmentally friendly alternatives to synthetic antioxidants [90].

### 6.2. Pharmaceutical Application

The area of the wound and the length of the epithelization phase significantly decreased during treatment with naringin ointment formulation, whilst the velocity of wound contraction dramatically increased. Naringin ointment formulation modulates collagen-1 expression to promote angiogenesis, which in turn promotes wound healing. This is accomplished by down-regulating the expression of inflammatory (ILs, NF-Jb, and TNF-a), apoptotic (pol-g and Bax), and growth factor (TGF-b and VEGF) genes [91]. An investigation was carried out into the potential protective benefits of hesperidin and naringin against diclofenac-induced liver damage and also the involvement of oxidative stress, inflammation, and apoptosis modulation. The study found that, when hesperidin and naringin were given to diclofenac-injected rats, the raised levels of blood LDH, GGT, ALP, AST, IL-17 levels, total bilirubin, and TNF level, liver p53 and caspase-3 mRNA expression, liver lipid peroxidation all significantly decreased. Through anti-inflammatory, antioxidant, and anti-apoptotic effects, hesperidin, naringin, and their combination proved effective for reversing diclofenac-induced liver damage. The liver adverse effects of medications like diclofenac can be treated with naringin, hesperidin, or a combination of the two [92].

### 6.3. In Livestock Sector

Naringin and quercetin reduce protozoa and methanogen populations in the rumen and suppress methane production without negatively affecting the parameters of ruminal fermentation. Daily diets containing hesperidin and naringin have proved successful in enhancing milk’s oxidative stability while having no negative impacts on the substance’s chemical compositions, coagulation abilities, or fatty acid profile [93]. According to studies, adding Macleaya cordata extract at a dosage of 120 mg/kg of diet and naringin at a dosage of 50 mg/kg of diet to post-weaning piglet diets enhanced growth performance and nutrient digestibility while having no effect on the villi and crypts’ histo-morphological status in the jejunum. These results show that these items have the potential to be utilized as feed supplements to improve the growth performance of weaned piglets [94]. Hesperidin and naringin, when consumed, had a substantial impact on the antioxidative capacity of broiler breast and thigh meat, presumably indicating that these bioflavonoids had entered the cell phospholipid membranes of the broiler muscles. Broiler meat’s slower lipid oxidation rate has a good impact on meat shelf life, which benefits both the consumer and the poultry meat industry. Hesperidin and naringin have a good effect on the antioxidative qualities of meat without having a negative impact on the development capacity and meat quality features of poultry; for this reason, they can be introduced as a significant additive to poultry feed [95].

### 6.4. Food Industry

The use of naringin microspheres in yogurt demonstrated their ability to effectively reduce whey precipitation and to slow pH drop. According to a study, naringin-encapsulated microspheres could extend the shelf life of this bioactive product and offer a fresh concept for functional yogurt [96]. Hesperidin, naringin, and coumarins have been found to inhibit xanthine oxidase, which directly reduces cellular free radical production. When compared to dietary citrus pulp and control diets, feeding dietary citrus pulp prolonged the shelf life of beef during retail display by increasing antioxidant activity, lowering coliforms, and reducing lipid and protein oxidation [97]. Naringin’s incorporation caused significant UV blocking, plasticizing, and antioxidant and antibacterial effects. The biological oxygen demand (BOD) in saltwater was used to test the biodegradability of these films, showing excellent disintegration under these circumstances [98]. It was observed that naringin could prevent browning in soybean and mung bean and could keep their appearance and quality intact for a period of six days while they were stored. Since naringin treatment enhanced the quantity of p-coumaric acid and gallic acid in mung bean sprouts while also increasing the quantity of rutin and daidzein in soybean sprouts, the utilization of naringin for the postharvest preservation of soybean sprouts and mung bean sprouts will maintain good consumer quality [99].

## 7. Conclusions and Future Prospects

Flavonoids are a large group of polyphenolic compounds that are present in almost every part of the plant, including the leaves, flowers, stems, roots, fruits, and seeds. Although flavonoids are classed differently in the study, they all share a similar structural foundation. Flavonoids are categorized into six different groups based on the variations in their substitution and the activity of the carbon skeletons: flavones, flavanones, flavan-3-ols, flavonols, anthocyanins, and isoflavones. There are almost 10,000 identified flavonoids, and several studies have shown their antioxidant, pro-oxidant, anti-inflammatory, antiviral/bacterial, antidiabetic, cardioprotective, anticancer, and anti-aging properties. Naringin (bitter flavonoid) has been reviewed in detail. Commercial grapefruit juice manufacturing can benefit from the use of the enzyme naringinase to get rid of the bitterness brought on by naringin. The stomach-based enzyme naringinase transforms naringin in humans into aglycone naringenin. Naringin has been demonstrated to possess anti-cancer, anti-apoptotic, cholesterol-lowering, anti-atherogenic, antioxidant, and metal binding properties. Naringin is also reported to improve the metabolism and absorption of medications. It has been reported that exposure to naringin in-vitro and in-vivo in a variety of test animals and cell lines has actions that may be used to treat osteoclast genesis, hyperthyroidism, hyperlipidemia, diabetes, tumors, and asthma. Recent research indicates that naringin may potentially be utilized to treat COVID-19. The value of naringin for both therapeutic and commercial applications has recently been the focus of greater investigation. Naringin is a promising research subject with several applications.

## Figures and Tables

**Table 1:** The major classes of flavonoids, examples, chemical structures, and main dietary sources.

Flavonoids	Examples	Chemical Structure with Molar Mass (g/mol)	Food Sources	Reference
Anthocyanin	Cyanidin, pelargonidin, peonidin	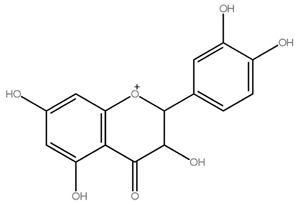 Cyanidin (287.24)	*Solanum melongena, Rubus fruticosus,* *Ribes nigrum, Vaccinium sect. Cyanococcus*	[15,16]
Flavan-3-ol	Catechin, epicatechin, epigallocatechin	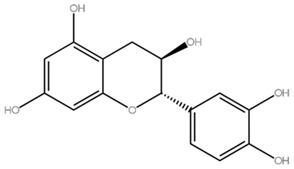 Catechin (290.26)	Green tea, Chocolate, *Phaseolus vulgaris* L., *Prunus avium*	[16]
Flavanones	Hesperidin, Naringin, Eriodictyol	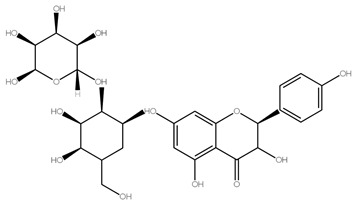 Naringin (580.54)	Orange juice, grapefruit juice, lemon juice	
Flavanones	Apigenin, luteolin	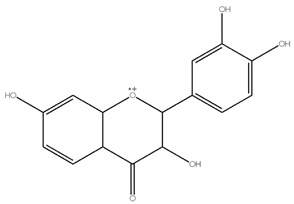 Apigenin (270.05)	*Petroselinum crispum, Apium graveolens, Capsicum annuum*	[17]
Flavonols	Quercetin, kaempferol, myricetin	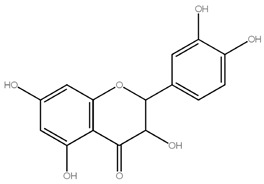 Quercetin (302.23)	*Allium cepa, Malus domestica, Brassica oleracea* var. *sabellica, Allium porrum*	[6]
Isoflavones	Genistein, daidzein, glycitein	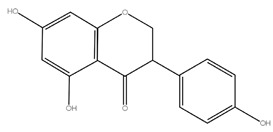 Genistein (270.24)	Soyflour, soymilk, *Glycine max.*	[18,19]

**Table 2:** Flavonoids isolated in citrus sp. their structure, molecular weight, fruit sources, C-ring structure and substitution pattern (FLA: flavanone FLO: flavone FOL: flavonol).

Flavonoid	Molecular Weight	C-Ring Structure	Fruit Sources	Substitution Pattern	Reference
Naringin	580.541 g/mol	FLA FLA	*Citrus paradisi* *Citrus aurantium*	5,4′-OH 7-O-Neo	[8,21]
Neoeriocitrin	596.5 g/mol	FLA	*Citrus aurantium*	5,3′,4′-OH 7-O-Neo	[6,8]
Diosmin	608.54 g/mol	FLO	*Citrus sinensis* *Citrus limonia*	5,3′-OH 4′-OMe 7-O-Rut	[29]
Hesperidin	610.1898 g/mol	FLA	*Citrus sinensis*	5,3′-OH, 4′-OMe 7-O-Rut	[28]
Rutin	610.517 g/mol	FOL	*Citrus limonia*	5,7,3′,4′-OH 3-O-Rut	[4,28]
Naringenin	272.257 g/mol	FLA	*Citrus paradisi*	5,7,4′-OH	[30,31]
Hesperetin	302.27 g/mol	FLA	*Citrus sinensis*	5,7,3′-OH 4′-OMe	[3,21]
Kaempferol	286.23 g/mol	FOL	*Citrus paradisi*	5,7,3,4′-OH	[8]
Quercetin	302.236 g/mol	FOL	*Citrus limonia*	5,7,3,3′,4′-OH	[28]
Tangeretin	372.37 g/mol	FLO	*Citrus aurantium* *Citrus paradisi* *Citrus limonia*	5,6,7,8,4′-OMe	[5]
Luteolin	286.24 g/mol	FLO	*Citrus limonia* *Citrus aurantium*	5,7,3′,4′-OH	[10]

**Table 3:** The therapeutic effect of naringin in different medical condition.

Medical Condition	Therapeutic Effects of Naringin	Other Possible Treatments	References
Diabetes	Improved insulin sensitivity, enhanced glucose uptake, inhibition of α-glucosidase enzyme, antioxidant, and anti-inflammatory properties.	Conventional antidiabetic medications, lifestyle modifications.	[5]
Cardiovascular Health	Reduction of cholesterol levels, prevention of LDL oxidation, anti-inflammatory effects, improvement of endothelial function.	Statins, blood pressure medications, lifestyle modifications.	[74]
Cancer	Anticancer properties, inhibition of tumor growth, induction of apoptosis, antioxidant and anti-inflammatory effects.	Chemotherapy, radiation therapy, targeted therapies.	[75]
Neurodegenerative Diseases	Neuroprotective effects, reduction of oxidative stress and inflammation, potential improvement of cognitive function.	Symptomatic treatments, rehabilitation therapies.	[76]
Liver Health	Protection against liver damage, reduction of liver fibrosis, antioxidant effects, improvement of liver enzyme levels.	Lifestyle modifications, hepatoprotective medications.	[77]
Obesity	Suppression of adipogenesis, reduction of body weight gain, improvement of metabolic parameters.	Diet and exercise interventions, weight loss medications.	[2]

## Data Availability

Not applicable.

## References

[1] Alam M.A., Subhan N., Rahman M.M., Uddin S.J., Reza H.M., Sarker S.D. (2014). Effect of citrus flavonoids, naringin and naringenin, on metabolic syndrome and their mechanisms of action. Adv. Nutr..

[2] Jakab J., Miškić B., Mikšić Š., Juranić B., Ćosić V., Schwarz D., Včev A. (2021). Adipogenesis as a potential anti-obesity target: A review of pharmacological treatment and natural products. Diabetes Metab. Syndr. Obes..

[3] Su W., Wang Y., Li P., Wu H., Zeng X., Shi R., Zheng Y., Li P., Peng W. (2020). The potential application of the traditional Chinese herb Exocarpium Citri grandis in the prevention and treatment of COVID-19. Tradit. Med. Res..

[4] Tang D.M., Zhu C.F., Zhong S.A., Da Zhou M. (2011). Extraction of naringin from pomelo peels as dihydrochalcone’s precursor. J. Sep. Sci..

[5] Ahmed O.M., Mahmoud A.M., Abdel-Moneim A., Ashour M.B. (2012). Antidiabetic effects of hesperidin and naringin in type 2 diabetic rats. Diabetol. Croat..

[6] Hollman P.C.H., Arts I.C.W. (2000). Flavonols, flavones and flavanols—Nature, occurrence and dietary burden. J. Sci. Food Agric..

[7] Gorinstein S., Leontowicz H., Leontowicz M., Krzeminski R., Gralak M., Delgado-Licon E., Martinez Ayala A.L.M., Katrich E., Trakhtenberg S. (2005). Changes in plasma lipid and antioxidant activity in rats as a result of naringin and red grapefruit supplementation. J. Agric. Food Chem..

[8] Li Q., Zhang N., Sun X., Zhan H., Tian J., Fei X., Liu X., Chen G., Wang Y. (2021). Controllable biotransformation of naringin to prunin by naringinase immobilized on functionalized silica. J. Chem. Technol. Biotechnol..

[9] Zeng X., Su W., Zheng Y., He Y., He Y., Rao H., Peng W., Yao H. (2019). Pharmacokinetics, tissue distribution, metabolism, and excretion of naringin in aged rats. Front. Pharmacol..

[10] Sharma A., Bhardwaj P., Arya S.K. (2021). Naringin: A potential natural product in the field of biomedical applications. Carbohydr. Polym..

[11] Egert S., Rimbach G. (2011). Which sources of flavonoids: Complex diets or dietary supplements. Adv. Nutr..

[12] Bacanli M., Başaran A.A., Başaran N. (2018). The major flavonoid of grapefruit: Naringin. Polyphenols: Prevention and Treatment of Human Disease.

[13] Renugadevi J., Prabu S.M. (2009). Naringenin protects against cadmium-induced oxidative renal dysfunction in rats. Toxicology.

[14] Panche A.N., Diwan A.D., Chandra S.R. (2016). Flavonoids: An overview. J. Nutr. Sci..

[15] Nawaz R., Abbasi N.A., Khan M.R., Ali I., Hasan S.Z.U., Hayat A. (2020). Color development in “Feutrell’s early” (Citrus reticulata Blanco) affects peel composition and juice biochemical properties. Int. J. Fruit Sci..

[16] Tan J., Li Y., Hou D. (2019). The Effects and Mechanisms of cyanidin-3-glucoside and Its phenolic Metabolites in Maintaining intestinal Integrity. Antioxidants.

[17] Puri M. (2012). Updates on naringinase: Structural and biotechnological aspects. Appl. Microbiol. Biotechnol..

[18] Křížová L., Dadáková K., Kašparovská J., Kašparovský T. (2019). Isoflavones. Molecules.

[19] Da Silva F.L., Escribano-Bailón M.T., Pérez Alonso J.J., Rivas-Gonzalo J.C., Santos-Buelga C. (2007). Anthocyanin pigments in strawberry. LWT—Food Sci. Technol..

[20] Addi M., Elbouzidi A., Abid M., Tungmunnithum D., Elamrani A., Hano C. (2022). An overview of bioactive flavonoids from citrus fruits. Appl. Sci..

[21] Verma A.K., Pratap R. (2012). Chemistry of biologically important flavones. Tetrahedron.

[22] Heiss C., Keen C.L., Kelm M. (2010). Flavanols and cardiovascular disease prevention. Eur. Heart J..

[23] Hackman R.M., Polagruto J.A., Zhu Q.Y., Sun B., Fujii H., Keen C.L. (2007). Flavanols: Digestion, absorption and bioactivity. Phytochem. Rev..

[24] Aherne S.A., O’Brien N.M. (2002). Dietary flavonols: Chemistry, food content, and metabolism. Nutr. J..

[25] Wollenweber E. (2017). Flavones and flavonols. The flavonoids. J. Adv. Res..

[26] Jung U.J., Kim S.R. (2014). Effects of naringin, A flavanone glycoside in grapefruits and citrus fruits, On the nigrostriatal dopaminergic projection in the adult brain. Neural Regen. Res..

[27] Felgines C., Texier O., Morand C., Manach C., Scalbert A., Régerat F., Remesy C., Felgines C., Texier O., Morand C. (2020). Bioavailability of the flavanone naringenin and its glycosides in rats. Am. J. Physiol. Gastrointest. Liver Physiol..

[28] Billowria K., Ali R., Rangra N.K., Kumar R., Chawla P.A. (2022). Bioactive Flavonoids: A Comprehensive Review on Pharmacokinetics and Analytical Aspects. Crit. Rev. Anal. Chem..

[29] Sharma K., Mahato N., Lee Y.R. (2019). Extraction, characterization and biological activity of citrus flavonoids. Rev. Chem. Eng..

[30] Zhang L., Song L., Zhang P., Liu T., Zhou L., Yang G., Lin R., Zhang J. (2015). Solubilities of naringin and naringenin in Different Solvents and Dissociation Constants of naringenin. J. Chem. Eng. Data.

[31] Aron P.M., Kennedy J.A. (2008). Flavan-3-ols: Nature, occurrence and biological activity. Mol. Nutr. Food Res..

[32] Ioannou I., Nouha M., Chaaban H., Boudhrioua N.M., Ghoul M. (2020). Effect of the process, temperature, light and oxygen on naringin extraction and the evolution of its antioxidant activity. Int. J. Food Sci. Technol..

[33] Wang S., Yang C., Tu H., Zhou J., Liu X., Cheng Y., Luo J., Deng X., Zhang H., Xu J. (2017). Characterization and metabolic diversity of flavonoids in Citrus species. Sci. Rep..

[34] Hoffmann E. (1879). Naringin (Hesperidin de Vry). Arch. Pharm..

[35] Liu Y., Heying E., Tanumihardjo S.A. (2012). History, global distribution, and nutritional importance of citrus fruits. Compr. Rev. Food Sci. Food Saf..

[36] Pichaiyongvongdee S., Haruenkit R. (2009). Comparative studies of limonin and naringin distribution in different parts of pummelo (*Citrus grandis* (L.) Osbeck) cultivars grown in Thailand. J. Nat. Sci..

[37] Sir K.A., Randa E., Amro A.B. (2018). Content of phenolic compounds and vitamin C and antioxidant activity in wasted parts of Sudanese citrus fruits. Food Sci. Nutr..

[38] Hassan R.A., Hozayen W.G., Abo Sree H.T., Al-Muzafar H.M., Amin K.A., Ahmed O.M. (2021). Naringin and hesperidin counteract diclofenac-induced hepatotoxicity in male Wistar rats via their antioxidant, anti-inflammatory, and antiapoptotic activities. Oxidative Med. Cell. Longev..

[39] Bahorun T., Ramful-baboolall D., Neergheen-bhujun V., Aruoma O.I.A., Verma S., Tarnus E., Robert C., Silva D., Rondeau P. (2012). Phytophenolic Nutrients in Citrus: Biochemical and Molecular Evidence. Advances in Citrus Nutrition.

[40] Zhao B.T., Kim E.J., Son K.H., Son J.K., Min B.S., Woo M.H. (2015). Quality evaluation and pattern recognition analyses of marker compounds from five medicinal drugs of Rutaceae family by HPLC/PDA. Arch. Pharm. Res..

[41] Grassino A.N., Pedisić S., Dragović-Uzelac V., Karlović S., Ježek D., Bosiljkov T. (2020). Insight into high-hydrostatic pressure extraction of polyphenols from tomato peel waste. Plant Foods Hum. Nutr..

[42] Victor M.M., David J.M., Sakukuma M.C.K., França E.L., Nunes A.V.J. (2018). A simple and efficient process for the extraction of naringin from grapefruit peel waste. Green Process. Synth..

[43] Chávez-González M.L., Sepúlveda L., Verma D.K., Luna-García H.A., Rodríguez-Durán L.V., Ilina A., Aguilar C.N. (2020). Conventional and emerging extraction processes of flavonoids. Processes.

[44] Ciğeroğlu Z., Bayramoğlu M., Kırbaşlar Ş.İ., Şahin S. (2021). Comparison of microwave-assisted techniques for the extraction of antioxidants from Citrus paradisi Macf. biowastes. J. Food Sci. Technol..

[45] Cheigh C.I., Chung E.Y., Chung M.S. (2012). Enhanced extraction of flavanones hesperidin and narirutin from Citrus unshiu peel using subcritical water. J. Food Eng..

[46] Yu J., Dandekar D.V., Toledo R.T., Singh R.K., Patil B.S. (2007). Supercritical fluid extraction of limonoids and naringin from grapefruit (Citrus paradisi Macf.) seeds. Food Chem..

[47] Giannuzzo A.N., Boggetti H.J., Nazareno M.A., Mishima H.T. (2003). Supercritical fluid extraction of naringin from the peel of Citrus paradisi. Phytochem. Anal..

[48] Liu Y., Zhang H., Yu H., Guo S., Chen D. (2019). Deep eutectic solvent as a green solvent for enhanced extraction of narirutin, naringin, hesperidin and neohesperidin from Aurantii Fructus. Phytochem. Anal..

[49] El Kantar S., Rajha H.N., Boussetta N., Vorobiev E., Maroun R.G., Louka N. (2019). Green extraction of polyphenols from grapefruit peels using high voltage electrical discharges, deep eutectic solvents and aqueous glycerol. Food Chem..

[50] Ma G., Zhang L., Sugiura M., Kato M. (2020). Citrus and Health. The Genus Citrus.

[51] Yan Y., Zhou H., Wu C., Feng X., Han C., Chen H., Liu Y., Li Y. (2021). Ultrasound-assisted aqueous two-phase extraction of synephrine, naringin, and neohesperidin from *Citrus aurantium* L. fruitlets. Prep. Biochem. Biotechnol..

[52] Gupta A.K., Dhua S., Sahu P.P., Abate G., Mishra P., Mastinu A. (2021). Variation in phytochemical, antioxidant and volatile composition of pomelo fruit (*Citrus grandis* (L.) osbeck) during seasonal growth and development. Plants.

[53] Ghanbari-Movahed M., Jackson G., Farzaei M.H., Bishayee A. (2021). A systematic review of the preventive and therapeutic effects of naringin against human malignancies. Front. Pharmacol..

[54] Li H., Yang B., Huang J., Xiang T., Yin X., Wan J., Luo F., Zhang L., Li H., Ren G. (2013). Naringin inhibits growth potential of human triple-negative breast cancer cells by targeting β-catenin signaling pathway. Toxicol. Lett..

[55] Chen R., Qi Q.L., Wang M.T., Li Q.Y. (2016). Therapeutic potential of naringin: An overview. Pharm. Biol..

[56] Cells N.S., Kim H., Song J.Y., Park H.J., Park H., Yun D.H., Chung J. (2009). Naringin protects against rotenone-induced apoptosis in human. J. Physiol. Pharmacol..

[57] Aroui S., Fetoui H., Kenani A. (2020). Natural dietary compound naringin inhibits glioblastoma cancer neoangiogenesis. BMC Pharmacol. Toxicol..

[58] Li J., Dong Y., Hao G., Wang B., Wang J., Liang Y., Liu Y., Zhen E., Feng D., Liang G. (2017). Naringin suppresses the development of glioblastoma by inhibiting FAK activity. J. Drug Target..

[59] Memariani Z., Abbas S.Q., ul Hassan S.S., Ahmadi A., Chabra A. (2021). Naringin and naringenin as anticancer agents and adjuvants in cancer combination therapy: Efficacy and molecular mechanisms of action, a comprehensive narrative review. Pharmacol. Res. Commun..

[60] Ramesh E., Alshatwi A.A. (2013). Naringin induces death receptor and mitochondria-mediated apoptosis in human cervical cancer (SiHa) cells. Food Chem. Toxicol..

[61] Ahmed S., Khan H., Aschner M., Hasan M.M., Hassan S.T.S. (2019). Therapeutic potential of naringin in neurological disorders. Food Chem. Toxicol..

[62] Zhou J., Xia L., Zhang Y. (2019). Naringin inhibits thyroid cancer cell proliferation and induces cell apoptosis through repressing PI3K/AKT pathway. Pathol. Res. Pract..

[63] Mahmoud A.M. (2016). Anti-diabetic effect of naringin: Insights into the molecular mechanism. Int. J. Obes..

[64] Pandey V.K., Shams R., Singh R., Dar A.H., Pandiselvam R., Rusu A.V., Trif M. (2022). A comprehensive review on clove (*Caryophyllus aromaticus* L.) essential oil and its significance in the formulation of edible coatings for potential food applications. Front. Nutr..

[65] Bharti S., Rani N., Krishnamurthy B., Arya D.S. (2014). Preclinical evidence for the pharmacological actions of naringin: A review. Planta Med..

[66] Chen F., Zhang N., Ma X., Huang T., Shao Y., Wu C., Wang Q. (2015). Naringin alleviates diabetic kidney disease through inhibiting oxidative stress and inflammatory reaction. PLoS ONE..

[67] Heidary Moghaddam R., Samimi Z., Moradi S.Z., Little P.J., Xu S., Farzaei M.H. (2020). Naringenin and naringin in cardiovascular disease prevention: A preclinical review. Eur. J. Clin. Pharmacol..

[68] Kumar R., Clermont G., Vodovotz Y., Chow C.C. (2004). The dynamics of acute inflammation. J. Theor. Biol..

[69] Lawrence T., Gilroy D.W. (2007). Chronic inflammation: A failure of resolution. Int. J. Exp. Pathol..

[70] El-Desoky A.H., Abdel-Rahman R.F., Ahmed O.K., El-Beltagi H.S., Hattori M. (2018). Anti-inflammatory and antioxidant activities of naringin isolated from Carissa carandas L.: In vitro and in vivo evidence. Phytomedicine.

[71] Shirani K., Yousefsani B.S., Shirani M., Karimi G. (2020). Protective effects of naringin against drugs and chemical toxins induced hepatotoxicity: A review. Phytother. Res..

[72] Pari L., Amudha K. (2011). Hepatoprotective role of naringin on nickel-induced toxicity in male Wistar rats. Eur. J. Clin. Pharmacol..

[73] Chen J.C., Li L.J., Wen S.M., He Y.C., Liu H.X., Zheng Q.S. (2011). Quantitative analysis and simulation of anti-inflammatory effects from the active components of Paino Powder in rats. Chin. J. Integr. Med..

[74] Yadav M., Sehrawat N., Singh M., Upadhyay S.K., Aggarwal D., Sharma A.K. (2020). Cardioprotective and hepatoprotective potential of citrus flavonoid naringin: Current status and future perspectives for health benefits. Asian J. Biol. Sci..

[75] Higashi Y. (2023). Endothelial Function in Dyslipidemia: Roles of LDL-Cholesterol, HDL-Cholesterol and Triglycerides. Cells.

[76] Kopustinskiene D.M., Jakstas V., Savickas A., Bernatoniene J. (2020). Flavonoids as anticancer agents. Nutrients.

[77] Franzoni F., Scarfò G., Guidotti S., Fusi J., Asomov M., Pruneti C. (2021). Oxidative stress and cognitive decline: The neuroprotective role of natural antioxidants. Front. Neurosci..

[78] Gillessen A., Schmidt H.H.J. (2020). Silymarin as supportive treatment in liver diseases: A narrative review. Adv. Ther..

[79] Kawaguchi K., Maruyama H., Hasunuma R., Kumazawa Y. (2011). Suppression of inflammatory responses after onset of collagen-induced arthritis in mice by oral administration of the Citrus flavanone naringin. Immunopharmacol. Immunotoxicol..

[80] Yang X., Zhao Y., Gu Q., Chen W., Guo X. (2022). Effects of naringin on postharvest storage quality of bean sprouts. Foods.

[81] Bailey D.G., Dresser G.K. (2004). Interactions between grapefruit juice and cardiovascular drugs. Am. J. Cardiol..

[82] Hsiu S.L., Huang T.Y., Hou Y.C., Chin D.H., Chao P.D. (2002). Comparison of metabolic pharmacokinetics of naringin and naringenin in rabbits. Life Sci..

[83] Tsai Y.J., Tsai T.H. (2012). Mesenteric lymphatic absorption and the pharmacokinetics of naringin and naringenin in the rat. J. Agric. Food Chem..

[84] Tesseromatis C., Alevizou A. (2008). The role of the protein-binding on the mode of drug action as well the interactions with other drugs. Eur. J. Drug Metab. Pharmacokinet..

[85] Zeng X., Yao H., Zheng Y., He Y., He Y., Rao H., Li P., Su W. (2020). Tissue distribution of naringin and derived metabolites in rats after a single oral administration. J. Chromatogr..

[86] Najmanová I., Vopršalová M., Saso L., Mladěnka P. (2020). The pharmacokinetics of flavanones. Crit. Rev. Food Sci. Nutr..

[87] Goyal A., Verma A., Dubey N., Raghav J., Agrawal A. (2022). Naringenin: A prospec-tive therapeutic agent for Alzheimer’s and Parkinson’s disease. J. Food Biochem..

[88] Turfus S.C., Delgoda R., Picking D., Gurley B.J. (2017). Pharmacokinetics. Pharmacognosy.

[89] Sudto K., Pornpakakul S., Wanichwecharungruang S. (2009). An efficient method for the largescale isolation of naringin from pomelo (*Citrus grandis*) peel. Int. J. Food Sci. Technol..

[90] Gollavilli H., Hegde A.R., Managuli R.S., Bhaskar K.V., Dengale S.J., Reddy M.S., Kalthur G., Mutalik S. (2020). Naringin Nano-ethosomal novel sunscreen creams: Development and performance evaluation. Colloids Surf. B Biointerfaces.

[91] Pandey V.K., Islam R.U., Shams R., Dar A.H. (2022). A comprehensive review on the application of essential oils as bioactive compounds in nano-emulsion based edible coatings of fruits and vegetables. Appl. Food Res..

[92] Goliomytis M., Kartsonas N., Charismiadou M.A., Symeon G.K., Simitzis P.E., Deligeorgis S.G. (2015). The influence of naringin or hesperidin dietary supplementation on broiler meat quality and oxidative stability. PLoS ONE.

[93] Oliva J., French B.A., Li J., Bardag-Gorce F., Fu P., French S.W. (2008). Sirt1 is involved in energy metabolism: The role of chronic ethanol feeding and resveratrol. Exp. Mol. Pathol..

[94] Lee S.H., Chow P.S., Yagnik C.K. (2022). Developing eco-friendly skin care formulations with microemulsions of essential oil. J. Cosmet. Sci..

[95] Simitzis P., Massouras T., Goliomytis M., Charismiadou M., Moschou K., Economou C., Papadedes V., Lepesioti S., Deligeorgis S. (2019). The effects of hesperidin or naringin dietary supplementation on the milk properties of dairy ewes. J. Sci. Food Agric..

[96] Goodarzi-Boroojeni F., Männer K., Zentek J. (2018). The impacts of Macleaya cordata extract and naringin inclusion in post-weaning piglet diets on performance, nutrient digestibility and intestinal histomorphology. Arch. Anim. Nutr..

[97] Kandhare A.D., Alam J., Patil M.V.K., Sinha A., Bodhankar S.L. (2016). Wound healing potential of naringin ointment formulation via regulating the expression of inflammatory, apoptotic and growth mediators in experimental rats. Pharm. Biol..

[98] Wang H., Hu H., Zhang X., Zheng L., Ruan J., Cao J., Zhang X. (2022). Preparation, physicochemical characterization, and antioxidant activity of naringin–silk fibroin–alginate microspheres and application in yogurt. Foods.

[99] Tayengwa T., Chikwanha O.C., Gouws P., Dugan M.E.R., Mutsvangwa T., Mapiye C. (2020). Dietary citrus pulp and grape pomace as potential natural preservatives for extending beef shelf life. Meat Sci..

